# Surface-Plasmon-Resonance-Based Optical-Fiber Micro-Displacement Sensor with Temperature Compensation

**DOI:** 10.3390/s18103210

**Published:** 2018-09-23

**Authors:** Yong Wei, Ping Wu, Zongda Zhu, Lu Liu, Chunlan Liu, Jiangxi Hu, Shifa Wang, Yonghui Zhang

**Affiliations:** 1 Chongqing Municipal Key Laboratory of Intelligent Information Processing and Control of Institutions of Higher Education, Chongqing Three Gorges University, Wanzhou, Chongqing 404100, China; weiyong@hrbeu.edu.cn; 2 College of Electronic & Information Engineering, Chongqing Three Gorges University, Wanzhou, Chongqing 404100, China; 20160040@sanxiau.edu.cn (P.W.); 20160008@sanxiau.edu.cn (J.H.); 20160010@sanxiau.edu.cn (S.W.); 3 National Key Laboratory of Science and Technology on Tunable Laser, Harbin Institute of Technology, Harbin 150001, China, 18b921014@stu.hit.edu.cn; 4 Department of Physics, Harbin Institute of Technology, Harbin 150001, China, 18b311002@stu.hit.edu.cn; 5 Chongqing Engineering Research Center of Internet of Things and Intelligent Control Technology, Chongqing Three Gorges University, Wanzhou, Chongqing 404100, China; guangxianchuangan@njust.edu.cn; 6 Basic Medicine Department, Chongqing Three Gorges Medical College, Wanzhou, Chongqing 404100, China

**Keywords:** fiber-optic sensors, surface plasmon resonance, displacement measurement, temperature compensation, micro-structure fiber

## Abstract

Micro-displacement measurements play a crucial role in many industrial applications. Aiming to address the defects of existing optical-fiber displacement sensors, such as low sensitivity and temperature interference, we propose and demonstrate a novel surface plasmon resonance (SPR)-based optical-fiber micro-displacement sensor with temperature compensation. The sensor consists of a displacement-sensing region (DSR) and a temperature-sensing region (TSR). We employed a graded-index multimode fiber (GI-MMF) to fabricate the DSR and a hetero-core structure fiber to fabricate the TSR. For the DSR, we employed a single-mode fiber (SMF) to change the radial position of the incident beam as displacement. The resonance angle in the DSR is highly sensitive to displacement; thus, the resonance wavelength of the DSR shifts. For the TSR, we employed polydimethylsiloxane (PDMS) as a temperature-sensitive medium, whose refractive index is highly sensitive to temperature; thus, the resonance wavelength of the TSR shifts. The displacement and temperature detection ranges are 0–25 μm and 20–60 °C; the displacement and temperature sensitivities of the DSR are 4.24 nm/μm and −0.19 nm/°C, and those of the TSR are 0.46 nm/μm and −2.485 nm/°C, respectively. Finally, by means of a sensing matrix, the temperature compensation was realized.

## 1. Introduction

Displacement sensors play a crucial role in many industrial applications, such as micro-manufacture, precise positioning, structure health monitoring, scanning tunneling microscopy, and so on. With the development of sensor technology, optical-fiber sensors became more popular due to their advantages over traditional electronic sensor systems, such as high sensitivity, instant response, immunity to electromagnetic interference, lower cost, compact structure, long-distance transmission, and so on. Until now, many optical-fiber displacement sensors have been proposed. The fiber Bragg grating (FBG) is a common way of fabricating displacement sensors [1,2]; however, their sensitivities are too low to measure micro-displacement accurately. By contrast, the displacement sensors based on long-period fiber gratings (LPFG) [3], optical-fiber interferometers (OFI) [4,5,6], single-mode–multimode–single-mode (SMS) fiber structures [7], and photonic crystal fiber (PCF) resonant cavities [8] have higher sensitivities and can measure micro-displacement on the order of micrometers or sub-micrometers, but their sensitivities still require improvement.

Surface plasmon resonance (SPR) optical-fiber sensors attracted much attention in recent decades. Compared with other optical-fiber sensors, SPR sensors have superior performance indicators, such as ultrahigh sensitivity and resolution, as well as simple and multifarious structures. SPR sensors are mainly applied to refractive index (RI) detection of chemical and biological analytes [9,10,11,12]. In the application field of displacement measurement, there are very few successful cases as well. J. Y. Lin et al. [13] and S. F. Wang et al. [14] proposed micro-displacement sensors using SPR heterodyne interferometry. Although their sensors have nano resolution, the sensor structure based on a prism is too large to integrate. Later, X. M. Wang et al. [15] proposed an optical-fiber micro-displacement sensor based on an Otto SPR configuration, where the air gap is used as displacement. This sensor has an ultrahigh sensitivity of 31.45 nm/nm and a pico resolution; however, due to the shortcomings of the Otto SPR configuration, the detection range of this sensor is ultra-narrow (0–10 nm) and the environmental interference is serious. By contrast, the Kretschmann SPR configuration is more popular due to its advantages of high sensitivity, wide detection range, and ease of integration into optical fiber. Last year, we proposed an optical-fiber micro-displacement sensor based on the Kretschmann SPR configuration [16]. Although the performance is good, regrettably, the temperature variation causes misdetection of displacement, which is a very serious defect.

In response to these issues, in this paper, we propose and demonstrate an SPR-based optical-fiber micro-displacement sensor with temperature compensation. We employed a graded-index multimode fiber (GI-MMF) to fabricate the displacement-sensing region (DSR) and a hetero-core structure fiber to fabricate the temperature-sensing region (TSR). For the DSR, we employed a single-mode fiber (SMF) to change the radial position of the incident beam as displacement. The resonance angle in the DSR is highly sensitive to displacement; thus, the resonance wavelength of the DSR shifts. For the TSR, we employed polydimethylsiloxane (PDMS) as a temperature-sensitive medium, whose RI is highly sensitive to temperature; thus, the resonance wavelength of the TSR shifts. Finally, by means of a sensing matrix, the temperature compensation was realized. To the best of our knowledge, this is the first time a temperature compensation optical-fiber micro-displacement sensor was realized using SPR technology, and its sensitivity is higher than almost all other optical-fiber displacement sensors.

## 2. Sensor Structure Design and Sensing Principle

### 2.1. Sensor Structure

**Figure 1:**
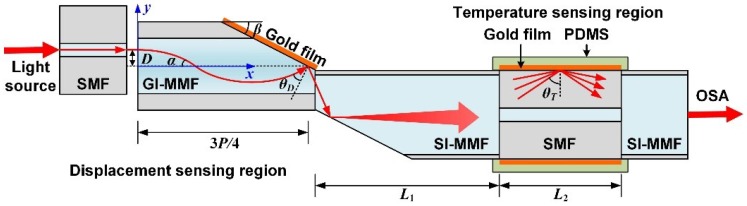
Sketch diagram (sectional view) of the micro-displacement optical-fiber sensor with temperature compensation.

Figure 1 shows the sketch diagram of the optical-fiber micro-displacement sensor with temperature compensation, which consists of the displacement-sensing region (DSR) and the temperature-sensing region (TSR). For the DSR, we fabricated a novel micro-structure optical fiber which was spliced by a short-section GI-MMF and a step-index multimode fiber (SI-MMF). The ends of both fibers were polished into a slope with the same angle of *β*. Then, a layer of gold film with a thickness of 50 nm was plated onto the polished surface of the GI-MMF for exciting surface plasmon waves. We employed an SMF to launch light beams from a white-light source into the GI-MMF; the SMF can be moved in the *y*-direction. Due to the special RI distribution of the GI-MMF, the beam propagation path can be approximately expressed as

(1) y=Dcos(2πx/P),

where *D* is the offset value between the axis of the SMF and GI-MMF, and *P* is the beam propagation period in the GI-MMF. By deriving Equation (1), we can get the included angle *α* between the light beam and the axis of the GI-MMF, which can be expressed as

(2) α=|arctan(2πD/P)|.

Therefore, we can get the included angle between the light beam and the gold film, which can be expressed as *α* + *β*. In other words, the incident angle *θ_D_* = 90° − (*α* + *β*), which changes with *D*; thus, the resonance wavelength of the SPR dip will also shift with *D*. In order to excite SPR exactly at the intersection of the fiber axis and the polished surface of GI-MMF, the length of the GI-MMF needs to be slightly larger than 3*P*/4. In addition, the DSR needs to be immersed in water.

For the TSR, we employed the well-known hetero-core fiber structure which consists of two SI-MMFs spliced to the ends of a conventional SMF with length *L*_2_ = 15 mm. Since the core diameter of the SMF is much smaller than that of the SI-MMF, and the light beam in the left-side SI-MMF is divergent, most of the light waves coming from the left-side SI-MMF will leak into the cladding of the SMF and exhibit total internal reflection on the cladding surface of the SMF. We plated a layer of gold film with a thickness of 50 nm on the cladding surface of SMF for exciting SPR. Then, we coated the SMF with a layer of PDMS which was used as a temperature-sensitive medium. When temperature changes, the RI of PDMS will also change significantly, which leads to a shift of the resonance wavelength of the SPR dip. Finally, the light beam which brings sensing information will re-couple into the right-side SI-MMF and be collected by the optical spectrum analyzer (OSA).

The light beam propagating in the cladding of the SMF has numerous modes; in other words, the incident angle is divergent between the critical angle and 90°. However, according to M. Iga [17], only a very narrow angle range plays a significant role for SPR excitation. To simplify analysis, we can assume the incident angle is a single value, *θ_T_*. In addition, for making the DSR and the TSR as close as possible, while making the incident angle *θ_T_* as unaffected by *D* as possible, we took the length of the left-side SI-MMF *L*_1_ as 25 mm.

### 2.2. Propagation Characteristics of the Light Beam in the GI-MMF

**Figure 2:**
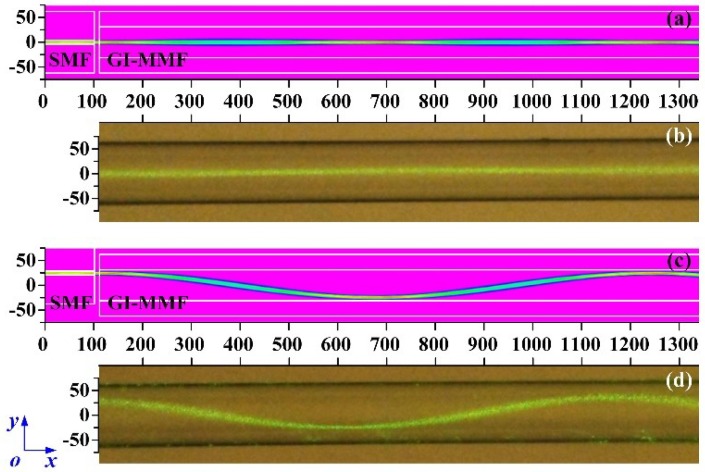
The simulated (**a**) and experimental (**b**) beam path in the graded-index multimode fiber (GI-MMF) under a displacement of 0 μm; the simulated (**c**) and experimental (**d**) beam path in the GI-MMF under a displacement of 25 μm.

In order to observe the beam path in the GI-MMF and measure the beam path period *P*, we performed simulations and experiments, respectively. The BeamPROP commercial software was employed to simulate the beam path with different *D*. The simulation conditions were as follows: the wavelength of the light source was 532 nm; the core and cladding diameters of the SMF were 8.2 μm and 125 μm, respectively; the RIs of the core and cladding of the SMF were 1.4682 and 1.4658 respectively; the length of the SMF was 100 μm; the core and cladding diameters of the GI-MMF were 62.5 μm and 125 μm, respectively; and the RI distribution of the GI-MMF core was parabolic. Using a refractive index profiler (S14, Photon Kinetics, Beaverton, OR, USA), we measured the maximum RI of the GI-MMF core as 1.4807 and the minimum as 1.4591. The distance between the SMF and the GI-MMF is 10 μm. The simulation results are shown in Figure 2a,c. When *D* = 0 μm, the beam path is straight and its diameter is almost equal to the core diameter of the SMF. When *D* = 0 μm, the beam path is sinusoidal and the period is 1135 μm. Then, we employed an inverted biological microscope (XDS-1B, COIC, Chongqing, China) with a magnification of 100× to observe the beam path in the GI-MMF. A laser source with a wavelength of 532 nm and a power of 100 mW was coupled into the GI-MMF through an SMF with the axis offset value *D*. In order to clearly observe the beam path, we placed the GI-MMF in water and weakened the background light of the microscope. When *D* = 0 μm, the beam path is straight, as also seen in the simulation (see Figure 2b); when *D* = 25 μm, the beam path is sinusoidal, as also seen in the simulation (see Figure 2d), and we measured the period *P* = 1075 μm, which was almost equal to the simulation result.

### 2.3. Temperature Characteristics of the PDMS and Water

**Figure 3:**
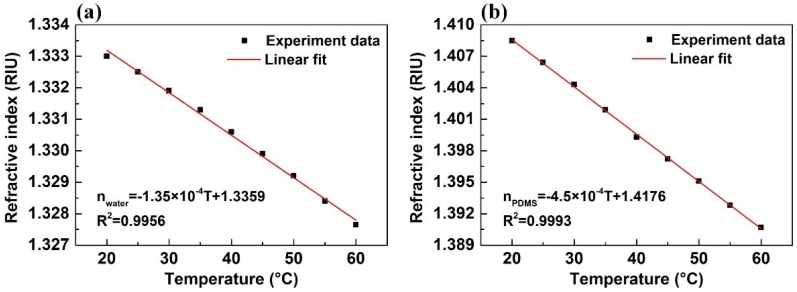
(**a**) The refractive index of water as a function of temperature; (**b**) the refractive index of polydimethylsiloxane (PDMS) as the function of temperature.

Both the refractive indices of water and PDMS change significantly with temperature. Using an Abbe refractometer, we measured the RI of water *n*_water_ and the RI of PDMS *n*_PDMS_ as a function of temperature *T* from 20 °C to 60 °C, with an interval of 5 °C; the test results are shown in Figure 3. The correlation curves are almost linear, and by linear fit, they can be expressed as follows:

(3) $$n_{\mathrm{water}}=-1.35\times 10^{-4}T+1.3359;$$

(4) $$n_{\mathrm{PDMS}}=-4.50\times 10^{-4}T+1.4176.$$

### 2.4. Spectrum Simulation

Then, we simulated and calculated the SPR transmitted spectra of the proposed optical-fiber micro-displacement sensor using the Fresnel formula [18]. The transmittance of the DSR (*R_D_*) and the TSR (*R_T_*) can be both expressed as

(5) $$R_{D,T}={\left|\left(r_{01}+r_{12}e^{2ik_{z}h}\right)/\left(1+r_{01}r_{12}e^{2ik_{z}h}\right)\right|}^{2},$$

where the subscripts *D* and *T* stand for the DSR and the TSR, respectively; the subscripts 0, 1, and 2 stand for the optical fiber, gold film, and the external medium, respectively; *h* is the thickness of the gold film; *r*_01_ is the reflection coefficient for the *p*-polarized incident beam at the interface between the optical fiber and the gold film; *r*_12_ is the reflection coefficient for the *p*-polarized incident beam at the interface between the gold film and the external medium; and *k_z_* is the *z*-component of the wave vector in the gold film. According to the Fresnel formula [18], they can be expressed as

(6) $$\begin{array}{l} r_{ik}=\frac{{\left(\varepsilon_{i}-\varepsilon_{0}\sin^{2}\theta_{D,T}\right)}^{0.5}/\varepsilon_{i}-{\left(\varepsilon_{k}-\varepsilon_{0}\sin^{2}\theta_{D,T}\right)}^{0.5}/\varepsilon_{k}}{{\left(\varepsilon_{i}-\varepsilon_{0}\sin^{2}\theta_{D,T}\right)}^{0.5}/\varepsilon_{i}+{\left(\varepsilon_{k}-\varepsilon_{0}\sin^{2}\theta_{D,T}\right)}^{0.5}/\varepsilon_{k}}\\ k_{z}=\left(2\pi /\lambda \right){\left(\varepsilon_{1}-\varepsilon_{0}\sin^{2}\theta_{D,T}\right)}^{0.5} \end{array}$$

where the subscripts *i*, *k* = 0, 1, 2, *θ* is the incident angle from the optical fiber to the gold film, *ε* is the dielectric constant, and *λ* is light wavelength. Finally, the total transmittance *R*_total_ of the sensor can be expressed as *R*_total_ = *R_D_* × *R_T_*.

**Figure 4:**
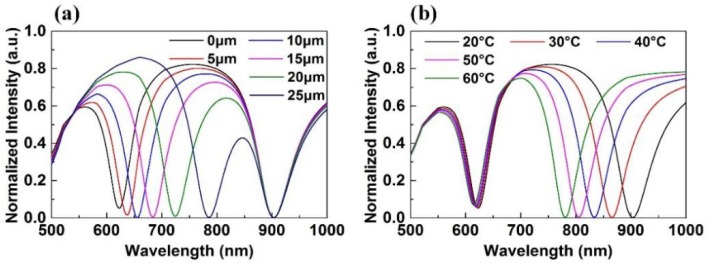
(**a**) Simulation result when *T* was fixed at 20 °C and *D* increased from 0 μm to 25 μm with intervals of 5 μm; (**b**) simulation result when *D* was fixed at 0 μm and *T* increased from 20 °C to 60 °C with intervals of 10 °C.

Combining Equations (2)–(6), we simulated and calculated the resonance wavelength as a function of displacement and temperature using the Matlab commercial software. The simulation conditions were as follows: the thickness of the gold film *h* = 50 nm, whose dielectric constant refers that used in Reference [19]. The polishing angle *β* was assumed to be 12° (i.e., *θ_D_* = 78° − *α*) and the incident angle *θ_T_* was assumed to be 82°. We executed two simulations: (1) a displacement simulation and (2) a temperature simulation. In the first simulation, the ambient temperature *T* was fixed at 20 °C, and displacement *D* increased from 0 μm to 25 μm with intervals of 5 μm. The simulation result is shown in Figure 4a. The first SPR dip is generated by the DSR, whose resonance wavelength red-shifts from 622.6 nm to 785.6 nm; thus, the average sensitivity is 6.52 nm/μm. The second SPR dip is generated by the TSR, whose resonance wavelength is unchanged at 903.3 nm. In the second simulation, displacement *D* was fixed at 0 μm, and the ambient temperature *T* increased from 20 °C to 60 °C with intervals of 10 °C. The simulation result is shown in Figure 4b. The resonance wavelength of the first SPR dip, which is generated by the DSR, blue-shifts from 622.6 nm to 614.5 nm; thus, the average sensitivity is −0.20 nm/°C. The resonance wavelength of the second SPR dip, which is generated by the TSR, blue-shifts from 903.3 nm to 780.7 nm; thus, the average sensitivity is −3.07 nm/°C. In addition, when the wavelength resolution of the OSA is 0.02 nm, the displacement and temperature resolutions are 3 nm and 6.5 × 10^−3^ °C, respectively.

## 3. Experimental Preparation

The simulation results prove that this sensor has a high displacement sensitivity, as well as temperature sensitivity. Correspondingly, we executed the experiments under the same conditions.

### 3.1. Fabrication of the Micro-Displacement Sensor

**Figure 5:**
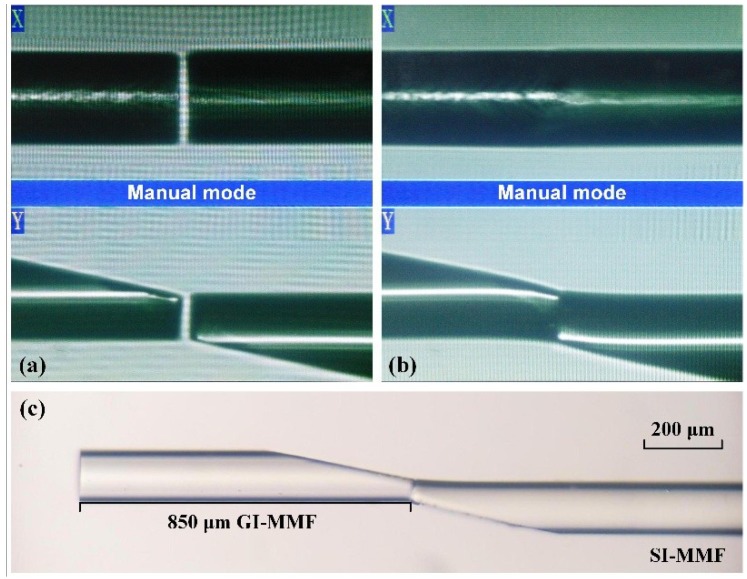
Photos during the fiber splicing process. (**a**) the GI-MMF tip (**left**) and the step-index multimode fiber (SI-MMF) tip (**right**) before splicing; (**b**) the GI-MMF tip (**left**) and the SI-MMF tip (**right**) after splicing, which had almost no deformation; (**c**) image of the fiber probe before film coating.

**Figure 6:**
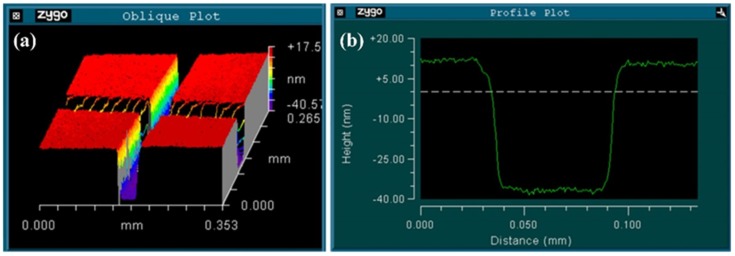
(**a**) Three-dimensional testing result for the gold coating with a groove; (**b**) test result for the groove depth, which is equal to the gold coating thickness.

We employed an optical-fiber fusion splicer (NT-400F, Notevio Toncom, Nanjing, China) to splice the SMF, GI-MMF, and SI-MMF. Firstly, we spliced the SMF to the right-side SI-MMF, and then cleaved to keep only an SMF section of length 15 mm. The core diameter of the used SI-MMF was 105 μm and its cladding diameter was 125 μm. Secondly, we spliced the left-side SI-MMF to an SMF section, and then cleaved to keep an SI-MMF section of length 25 mm. In the above two splicing processes, the fusion splicer worked in auto mode and the default splicing parameter was used. Thirdly, we polished the left-end SI-MMF with a polishing angle *β* = 12° using a fiber grinding and polishing machine (homemade, angle accuracy of 0.1° and depth accuracy of 1 μm) [20]; meanwhile, we polished the GI-MMF with the same angle. During the fiber-polishing process, we ground the fibers with 8000-grit grinding paper for 2 h, and then, we polished them with 12,000-grit polishing paper to make sure the ground surface was completely flat. Fourthly, we spliced the polished GI-MMF and the polished SI-MMF with reversed shape. In this splicing process, the fusion splicer worked in manual mode. We rotated and moved both fibers to arrange them in the wanted position (as shown in Figure 5a), and then fused both fibers with suitable fusing parameters (splicing intensity of 30 and splicing time of 0.8 s). The splicing spot is shown in Figure 5b; we can see that the optical fiber tips had almost no deformation after fusion splicing. In addition, our previous works [21,22] proved that this fiber splicing structure (we call it butt-joint) has good strength and stability. Moreover, this butt-joint technology is so mature that the fiber splicing structure has good reproducibility. Fifthly, we cleaved the GI-MMF and kept only a GI-MMF section of length 850 μm, which was slightly larger than 3*P*/4. The photomicrograph of the GI-MMF after cleaving is shown in Figure 5c. However, regrettably, due to our current cleaving technology with low accuracy (±10 μm), we cannot repeat this process with a high accuracy (we repeated 10 times and only one fiber was exactly 850 μm). Sixthly, we plated the gold film on the polished surface of the GI-MMF and the surface of the SMF section using a plasma sputtering apparatus (JS-1600, Beijing HTCY Technology, Beijing, China). During the latter plating process, we installed a rotating fixture in the plasma sputtering apparatus for fixing the fiber and plating the annular gold film. We employed a three-dimensional morphology analyzer (NewView7200, Zygo, Middlefield, CT, USA) to evaluate the gold coating quality. The gold film was scratched in a cross-shape groove. The flatness and the depth of the gold coating can be observed in Figure 6a,b. The depth of the groove indicates the thickness of the gold coating, which was 50 nm. Finally, we coated the SMF section with PDMS, whose thickness was about 1 mm. The PDMS coating technology was described in our earlier work [23].

### 3.2. Experimental Set-Up

**Figure 7:**
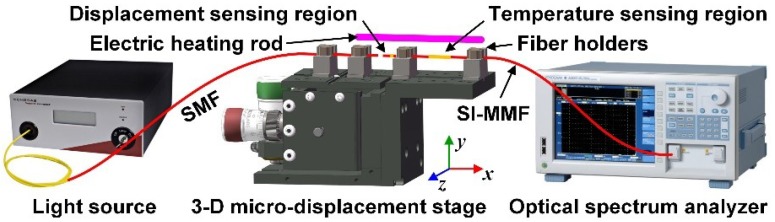
Sketch diagram of the experiment setup.

The experiment system is shown in Figure 7. A three-dimensional micro-displacement stage (MBT621D/M, Thorlabs, NJ, USA), whose accuracy is 1 μm, was employed to fix the fiber probe and move the SMF. A long-strip electric heating rod was placed close to the fiber sensing probe for changing the ambient temperature, and a thermometer, whose accuracy is 0.1 °C, was used to monitor the temperature in real time. The light beam from the supercontinuum light source (SuperK compact, NKT Photonic, Birkerød, Denmark), whose wavelength range is 450 nm to 2400 nm, and whose maximum power is 1.2 μW, was launched into the GI-MMF with radial offset value *D* to excite SPR phenomenon, and the transmitted beams were received by an optical spectrum analyzer (AQ6373, Yokogawa, Tokyo, Japan), whose detection range is 350 nm to 1200 nm, and whose wavelength resolution is 0.02 nm. The DSR was immersed in water. The maximum power of the transmitted beam was about 0.5 μW; thus, the insertion loss of the displacement sensor was about 3.8 dB.

## 4. Results and Discussion

### 4.1. Displacement Experiment

**Figure 8:**
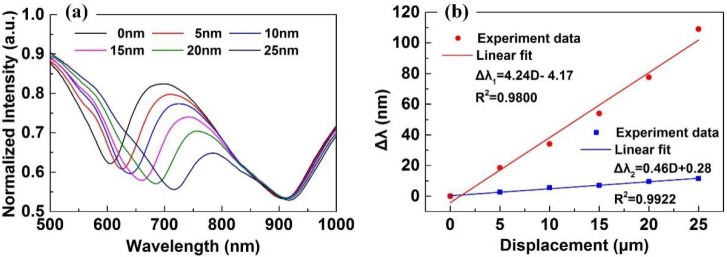
(**a**) The experimental surface plasmon resonance (SPR) spectrum changing with *D*; (**b**) the resonance wavelength shift Δ*λ* of the displacement-sensing region (DSR; red line) and the temperature-sensing region (TSR; blue line) as a function of *D*.

In this experiment, the ambient temperature *T* was fixed at 20 °C, and displacement *D* increased from 0 μm to 25 μm with intervals of 5 μm. In order to normalize all of the experiment spectra, we collected the spectrum of the light source as the reference spectrum. In addition, we smoothed the spectra using the smooth function of MatLab. The experimental results are shown in Figure 8. As shown in Figure 8a, the first SPR dip is generated by the DSR, whose resonance wavelength red-shifts from 606.0 nm to 715.0 nm. The second SPR dip is generated by the TSR, whose resonance wavelength red-shifts from 903.6 nm to 915.0 nm. In addition, comparing with the simulation results in Figure 4, the minimum of the experimental SPR dip is not smaller than 0.5; the reason is that only the *p*-polarized beam in the fiber can excite SPR, while the *s*-polarized beam cannot, and each of them has half of the energy. Figure 8b shows the resonance wavelength shift Δ*λ* of the DSR (red line) and the TSR (blue line) as a function of *D*. By linear fit, we can get that the displacement sensitivities of the DSR and the TSR were 4.24 nm/μm (*R^2^* = 0.9800) and 0.46 nm/μm (*R^2^* = 0.9922), respectively.

Comparing with the simulation results in Figure 4, the experimental displacement sensitivity of the DSR is quite low. The reason is that we do not consider the divergence angle of the light beam in the GI-MMF in the simulations; thus, the incident angle *θ_D_* is a single value (i.e., *θ_D_* = 78° − |arctan(2π*D*/*P*)|). However, in fact, the light beam propagating in the GI-MMF is slightly diverging with a small divergence angle *δ*; thus, the incident angle *θ_D_* is a range value (i.e., *θ_D_* = 78° − |arctan(2π*D*/*P*)| ± *δ*). As a result, the experimental spectra have a wider full width at half maximum (FWHM), while the experimental sensitivity is also reduced. In particular, with increasing *D*, the *δ* also increases; thus, the range of *θ_D_* widens, leading to a larger difference between experimental and simulation results. If we could measure the divergence angle *δ* and consider it in the simulations, the difference between simulations and experiments would be reduced. Moreover, in the experiment, the resonance wavelength of the TSR was not unchanged. The reason is that, with decreasing of *θ_D_*, the incident angle *θ_T_* also decreases slightly. However, we have no idea of how to calculate the slight change in *θ_T_*; thus, we assumed it to be invariable in the simulations.

**Figure 9:**
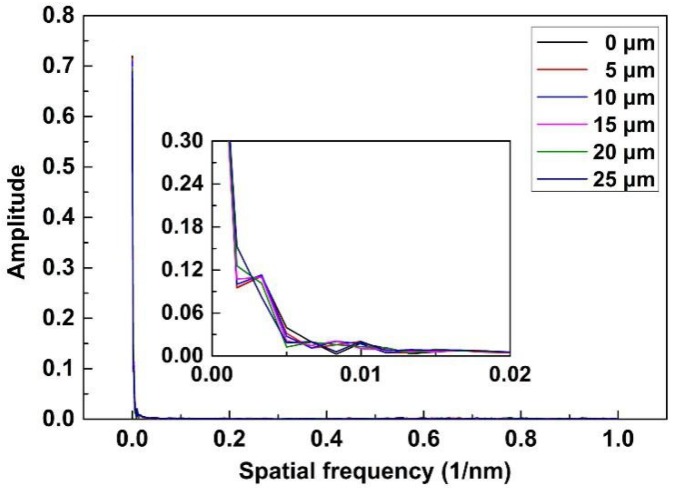
Fast Fourier transform (FFT) results of the original and unsmoothed SPR transmitted spectra.

Due to the sensor structure formed by splicing different types of fibers, it is able to generate intermodal interference. The intermodal interference spectrum can be detected with fast Fourier transform (FFT) [24,25]. Figure 9 shows the FFT results of the original and unsmoothed SPR transmitted spectra. Only some peaks at spatial frequencies between 0 and 0.01 nm^−1^ exist. Using the FFT filter, we find that these peaks make up the smoothed SPR spectra. In addition, because there are no any other peaks, the intermodal interference is not generated in the entire sensing head.

### 4.2. Temperature Experiment

**Figure 10:**
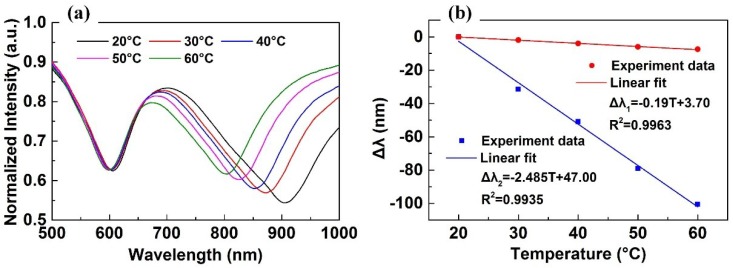
(**a**) The experimental SPR spectrum changing with *T*; (**b**) the resonance wavelength shift Δ*λ* of DSR (red line) and TSR (blue line) as a function of *T*.

In this experiment, the displacement *D* was fixed at 0 μm, and the ambient temperature *T* increased from 20 °C to 60 °C with intervals of 10 °C. The experimental results are shown in Figure 10. As shown in Figure 10a, the resonance wavelength of the first SPR dip, which is generated by the DSR, blue-shifts from 606.0 nm to 598.6 nm, and the resonance wavelength of the second SPR dip, which is generated by the TSR, blue-shifts from 903.6 nm to 803.0 nm. Figure 10b shows the resonance wavelength shift Δ*λ* of the DSR (red line) and the TSR (blue line) as a function of *T*. By linear fit, we can get that the temperature sensitivities of the DSR and the TSR were −0.19 nm/°C (*R^2^* = 0.9963) and −2.485 nm/°C (*R^2^* = 0.9935), respectively.

The experimental result is almost identical to the simulation result, although the temperature sensitivities of the former are lower than the latter, and the FWHM values of the former are wider.

### 4.3. Temperature Compensation by Means of the Sensing Matrix

Changes in displacement (Δ*D*/μm) and ambient temperature (Δ*T*/°C) produced changes in the resonance wavelengths of the DSR (Δ*λ_D_*/nm) and the TSR (Δ*λ_T_*/nm); this relationship can be mathematically expressed by the following set of equations:

(7) $$\left[\begin{array}{c} \Delta \lambda_{D}\\ \Delta \lambda_{T} \end{array}\right]=\left[\begin{array}{cc} 4.24\pm 0.3 & -0.19\pm 0.005\\ 0.46\pm 0.02 & -2.485\pm 0.1 \end{array}\right]\left[\begin{array}{c} \Delta D\\ \Delta T \end{array}\right].$$

By solving the above equation, we can get the sensing matrix, which can be expressed as

(8) $$\left[\begin{array}{c} \Delta D\\ \Delta T \end{array}\right]=\left[\begin{array}{cc} 0.239\pm 0.02 & -0.018\mp 0.001\\ 0.044\mp 0.0006 & -0.406\pm 0.02 \end{array}\right]\left[\begin{array}{c} \Delta \lambda_{D}\\ \Delta \lambda_{T} \end{array}\right].$$

Therefore, by means of the sensing matrix, we can simultaneously measure displacement and temperature, and the temperature compensation is then realized. In addition, when resolution of the OSA is 0.02 nm, the displacement and temperature resolutions are 5 nm and 8 × 10^−3^ °C, respectively.

### 4.4. Discussion

Table 1 shows the parameter comparison between our sensor and other optical-fiber micro-displacement sensors based on FBGs, LPFGs, optical-fiber modal interferometers (OFMI), optical-fiber Mach–Zehnder interferometers (OFMZI), SMS structures, and Otto SPR structures. According to Table 1, it can be seen that our sensor has excellent performance. The displacement sensitivity of our sensor is 4.24 nm/μm, which is the maximum of these sensors except for that in Reference [15]. The displacement resolution of our sensor is 5 nm, which is the minimum of these sensors except for that in Reference [15]. However, the Otto-SPR-structure-based displacement sensor has serious temperature interference, and the displacement range is very narrow. In addition, the displacement range of the sensor is determined by the core diameter of the GI-MMF; thus, if we replace it with another GI-MMF with larger core diameter, the displacement range will increase.

## 5. Conclusions

In summary, we demonstrated a novel SPR-based optical-fiber micro-displacement sensor, which can measure displacement and temperature simultaneously. Thanks to the special light beam propagation pathway in the GI-MMF, we established the relationship between displacement and resonance angle. By polishing and splicing the GI-MMF and SI-MMF, and then coating gold film on the polishing surface, we fabricated a Kretschmann SPR configuration; then the displacement-sensing region was realized. With an increase in displacement, the resonance wavelength of the displacement-sensing region red-shifts. In addition, we fabricated the temperature-sensing region by employing a PDMS-coated hetero-core structure optical-fiber. With an increase in temperature, the refractive index of the PDMS decreases; thus, the resonance wavelength of the temperature-sensing region blue-shifts.

Comparing with other optical-fiber micro-displacement sensors based on FBG, LPFG, OFI, and other optical-fiber structures, the proposed sensor has excellent performance. The displacement detection range is wide, from 0 to 25 μm, and temperature detection range is 20–60 °C. The corresponding sensitivities are 4.24 nm/μm and −2.485 nm/°C, and the resolutions are 5 nm and 8 × 10^−3^ °C, respectively. By means of the sensing matrix, the temperature compensation was realized. Most of all, this research will promote and expand the application of SPR technology in the field of mechanical measurements.

## Figures and Tables

**Table 1:** Parameter comparison between different optical-fiber micro-displacement sensors. FBG—fiber Bragg grating; LPFG—long-period fiber grating; OFMI—optical-fiber modal interferometer; OFMZI—optical-fiber Mach–Zehnder interferometer; SPR—surface plasmon resonance.

Sensor Type	Sensitivity	Range	Resolution
FBG [2]	0.55 nm/mm	0–0.5 mm	36 μm
LPFG [3]	0.22 nm/μm	0–140 μm	90 nm
OFMI [4]	−0.1 nm/μm	0–30 μm	0.2 μm
OFMZI [5]	−1.533 nm/μm	0–80 μm	13 nm
SMS structure [7]	5.89 pm/μm	0–600 μm	3.4 μm
Otto-SPR structure [15]	31.45 nm/nm	0–10 nm	0.6 pm
This paper	4.24 nm/μm	0–25 μm	5 nm
